# Antenatal Screening for Hepatitis B Virus in Uganda: Missed Opportunities for Diagnosis and Treatment

**DOI:** 10.1093/ofid/ofae603

**Published:** 2025-03-10

**Authors:** Melanie Etti, Hannah G Davies, Alexander Amone, Mary Kyohere, Valerie Tusubira, Jessica Burt, Geraldine O’Hara, Godfrey Matovu, Joseph Peacock, Annettee Nakimuli, Philippa Musoke, Musa Sekikubo, Kirsty Le Doare, PROGRESS Study Author Group, Abdelmajid Djennad, Agnes Nyamaizi, Agnes Ssali, Alexander Amone, Amusa Wamawobe, Annettee Nakimuli, Caitlin Farley, Carol Nanyunja, Christine Najuka, Cleophas Komugisha, Dan R Shelley, Edward A R Portal, Ellie Duckworth, Emilie Karafillakis, Geraldine O’Hara, Godfrey Matovu, Hannah G Davies, Janet Seeley, Joseph Peacock, Juliet Nsimire Sendagala, Katie Cowie, Kirsty Le Doare, Konstantinos Karampatsas, Lauren Hookham, Madeleine Cochet, Margaret Sewegaba, Mary Kyohere, Maxensia Owor, Melanie Etti, Merryn Voysey, Moses Musooko, Musa Sekikubo, Owen B Spiller, Patience Atuhaire, Paul T Heath, Philippa Musoke, Phiona Nalubega, Pooja Ravji, Richard Katungye, Ritah Namugumya, Rosalin Parks, Rose Azuba, Sam Kipyeko, Simon Beach, Stephen Bentley, Tim Old, Tobius Mutabazi, Valerie Tusubira, Vicki Chalker

**Affiliations:** Institute for Infection and Immunity, St George's, University of London, London, United Kingdom; Makerere University–Johns Hopkins University Research Collaboration, Kampala, Uganda; Institute for Infection and Immunity, St George's, University of London, London, United Kingdom; Makerere University–Johns Hopkins University Research Collaboration, Kampala, Uganda; Faculty of Infectious and Tropical Diseases, London School of Hygiene and Tropical Medicine, London, United Kingdom; Makerere University–Johns Hopkins University Research Collaboration, Kampala, Uganda; Institute for Infection and Immunity, St George's, University of London, London, United Kingdom; Makerere University–Johns Hopkins University Research Collaboration, Kampala, Uganda; Makerere University–Johns Hopkins University Research Collaboration, Kampala, Uganda; Makerere University–Johns Hopkins University Research Collaboration, Kampala, Uganda; Faculty of Infectious and Tropical Diseases, London School of Hygiene and Tropical Medicine, London, United Kingdom; Medical Research Council/Uganda Virus Research Institute and London School of Hygiene and Tropical Medicine Uganda Research Unit, Entebbe, Uganda; Department of Obstetrics and Gynecology, School of Medicine, Makerere University, Kampala, Uganda; Institute for Infection and Immunity, St George's, University of London, London, United Kingdom; Medical Research Council/Uganda Virus Research Institute and London School of Hygiene and Tropical Medicine Uganda Research Unit, Entebbe, Uganda; Makerere University–Johns Hopkins University Research Collaboration, Kampala, Uganda; Medical Research Council/Uganda Virus Research Institute and London School of Hygiene and Tropical Medicine Uganda Research Unit, Entebbe, Uganda; Institute for Infection and Immunity, St George's, University of London, London, United Kingdom; Makerere University–Johns Hopkins University Research Collaboration, Kampala, Uganda; Medical Research Council/Uganda Virus Research Institute and London School of Hygiene and Tropical Medicine Uganda Research Unit, Entebbe, Uganda

**Keywords:** antenatal care, antenatal screening, hepatitis B infection, hepatitis B surface antigen, hepatitis B virus

## Abstract

**Background:**

Hepatitis B virus (HBV) infection is a significant cause of morbidity and mortality globally. The World Health Organization estimates that just 10.5% of individuals living with HBV globally are aware of their status. Antenatal care provides an opportunity to screen pregnant women for HBV and to treat those who are eligible to reduce the risk of vertical transmission. We conducted an observational study to determine the proportion of pregnant women with active HBV infection delivering at a government-funded hospital in Kampala, Uganda, to estimate the number of missed opportunities to prevent vertical transmission.

**Methods:**

Eligible participants were enrolled via the PROGRESS study, an observational cohort study undertaken in Kampala, Uganda, between November 2018 and April 2021. Results presented here describe data from April 2019 to November 2020. Five milliliters of venous blood was drawn shortly after delivery. Serum aliquots were analyzed for hepatitis B surface antigen (HBsAg). HBsAg-positive participants were informed of their result by telephone and referred to the gastroenterology service for specialist management.

**Results:**

In total, 6062 women were enrolled between April 2019 and November 2020. Results were available for 6012 (99.6%) participants, among whom 131 (2.2%) were HBsAg positive. Only 10 of 131 (7.6%) HBsAg-positive participants were successfully referred to the gastroenterology service at Mulago Hospital for treatment of their infection.

**Conclusions:**

Our study identified a number of missed opportunities to identify active HBV infection among our pregnant cohort. Additional resources are urgently required to increase the coverage of antenatal HBV screening while also improving treatment pathways for pregnant women with HBV infection in this region.

## Teaser

We determined the proportion of pregnant women with active hepatitis B virus (HBV) infection at the moment of delivery in a government-funded hospital in Uganda, and identified a number of missed opportunities to identify HBV infection antenatally and prevent vertical transmission.

Hepatitis B virus (HBV) infection is a major public health problem globally. HBV infection causes both acute and chronic hepatitis and cirrhosis, and is a leading cause of hepatocellular carcinoma (HCC) worldwide, accounting for 749 000 new HCC cases and 692 000 HCC-related deaths annually [1, 2]. The latest global surveillance published in 2022 estimated that around 316 million people worldwide are living with HBV infection, although the World Health Organization (WHO) estimates that only 10.5% of those living with the disease are aware of their status [3, 4].

HBV can be transmitted through contact with infected blood, semen, or vaginal fluid or vertically from mother to child. Mother-to-child transmission (MTCT) of HBV is one of the most common routes of disease transmission worldwide and is considered the main driver of endemicity of chronic HBV infection in areas with intermediate to high disease prevalence [5]. The risk of chronic HBV infection is inversely correlated with the age of infection, meaning infants infected during the perinatal period are significantly more likely to develop chronic infection than those infected later in life [6]. Approximately 80%–90% of infants infected with HBV during their first year of life will develop chronic disease, compared to 30%–50% of children who are infected during early childhood before age 6, and 5%–10% of people who are infected during adulthood [7, 8].

The WHO estimates that approximately 70% of new HBV infections each year occur in Africa, where the burden of disease is already exceedingly high [9]. Around 81 million people in Africa are chronically infected with HBV, which includes 4.5 million children aged <5 years (70% of the global cases among children in this age group) [9]. While birth dose HBV vaccination and immune globulin are considered critical interventions for reducing the risk of MTCT of HBV [10], MTCT of HBV still occurs in at least 10% of exposed infants born to women with high-level HBV viremia despite appropriate immunoprophylaxis [11]. As such, antenatal testing for HBV infection and peripartum antiviral prophylaxis for pregnant women remain important strategies for reducing the risk of MTCT of HBV [12].

Despite the urgent need to expand HBV testing during pregnancy, antenatal screening for HBV is not routinely performed in most low-resource countries, including Uganda. The Ministry of Health of Uganda recommends that pregnant women be tested for hepatitis B surface antigen (HBsAg) at their first (ideally before 20 weeks' gestation) and second (ideally between 20–28 weeks' gestation) antenatal visits [13]; however, this has not yet been adopted as standard of care in most government healthcare facilities in the country. We conducted an observational study, nested within a large cohort study, to determine the prevalence of HBsAg among pregnant women delivering at a government-funded hospital in Kampala, Uganda, to estimate the potential number of missed opportunities to prevent vertical transmission of this disease.

This article forms part of a supplement based on the Progressing Group B Streptococcal Vaccines (PROGRESS) study. The PROGRESS study aimed to describe the causes of infectious mortality and morbidity in pregnancy and neonates, as well as the seroepidemiology of group B streptococcal infection—the major cause of neonatal sepsis worldwide—in Kampala, Uganda [14].

## METHODS

### Recruitment and Study Procedures

Pregnant women were recruited between April 2019 and March 2020 from Kawempe National Referral Hospital via the PROGRESS study (NCT04549220). Detailed information regarding recruitment methods, research protocol, and results has been published separately [14]. A 5-mL sample of venous blood was then taken from women presenting in labor who provided written informed consent, which was used to perform serological hepatitis B and human immunodeficiency virus (HIV) testing. Pre- and post-test counseling was provided for all enrolled participants. Demographic information was collected on the participant as well as the details of the delivery, birth outcomes of the infant, and the mid-upper arm circumference (MUAC). Participants who had a positive HBsAg result were informed of their results by telephone and referred to the gastroenterology service at Mulago National Referral Hospital for specialist management. Participant recruitment sites for the studies that form part of this supplement are detailed in a flowchart available in the supplementary material of another paper published in this issue [15].

### Laboratory Methods

Venous blood samples were centrifuged at Kawempe National Referral Hospital within 12 hours of collection to separate serum from plasma, and then transferred at room temperature to the Medical Research Council/Uganda Virus Research Institute and the London School of Hygiene and Tropical Medicine Uganda Research Centre for analysis. Sera extracted from blood samples were separated to aliquots of 1 mL and stored at -70°C. Following completion of recruitment, the frozen serum samples were defrosted and analyzed for the presence of HBsAg using the Roche Elecsys HBsAg II immunoassay within the Roche Diagnostics cobas 6000 analyzer (Roche Molecular Systems, Branchburg, New Jersey). A positive HBsAg result was defined as a sample with cut-off index ≥1.0 [16].

### Statistical Analyses

To describe demographic and other characteristics of the participants, mean and standard deviation (SD) were presented for normally distributed data and median and interquartile range (IQR) for non-normally distributed data. Point estimates were calculated for the prevalence of HBsAg seropositivity along with associated 95% confidence intervals (CIs). Binomial data were presented with percentages and 95% CIs. Measures of association for non-parametric data were conducted using the χ^2^ test.

## RESULTS

### Description of the Study Population

**Figure 1.**
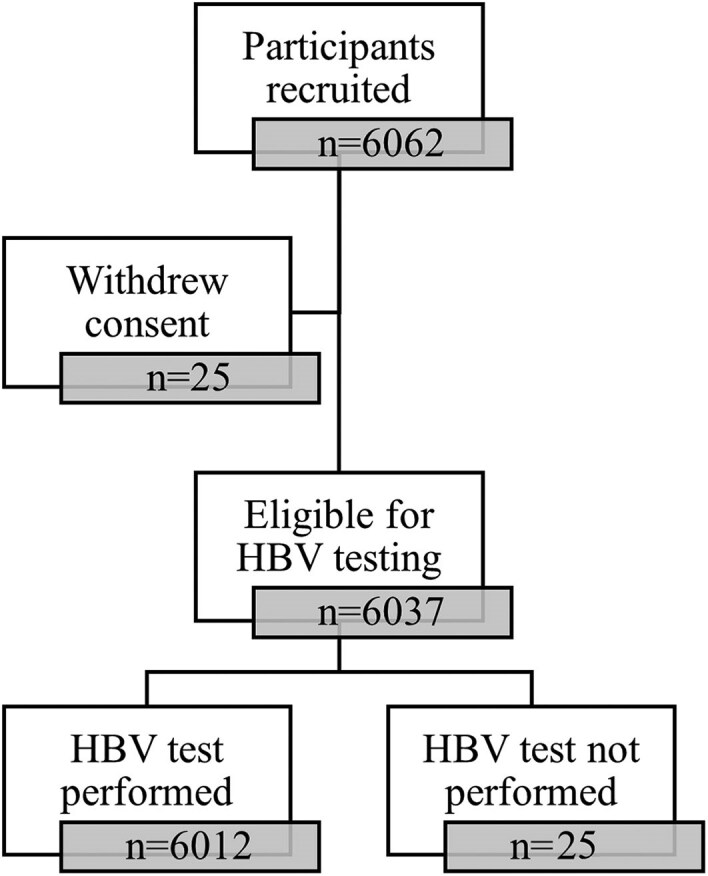
Flowchart of participant recruitment and hepatitis B virus (HBV) surface antigen testing among pregnant women enrolled to the PROGRESS study.

A total of 6062 women were recruited into the study: 25 withdrew consent, leaving 6037 in the cohort for analysis (Figure 1). The demographic, obstetric, and medical characteristics of enrolled participants is shown in Table 1. The mean age of the women enrolled in the study was 25.7 years (SD, 5.7 years). The majority of the women enrolled were Ugandan (98.6%) with a small number of participants from Rwanda, Kenya, Democratic Republic of Congo, Tanzania, and South Sudan. Most participants enrolled in the study had some formal education (94.9%); 5.1% of participants had never attended school, while 28% had a primary education only. Approximately 34% of women were recruited during their first pregnancy. The median gravidity of the participants was 2 (IQR, 2–4), and 208 (3.4%) women had an MUAC <23 cm.

**Table 1.** Demographic, Obstetric, and Medical Characteristics of PROGRESS Study Participants Tested for Hepatitis B Surface Antigen

Characteristic	No. (%)
Age, y	
14–18	371 (6.2)
19–23	2112 (35.0)
24–28	1817 (30.1)
>28	1736 (28.8)
Total	6037
Single/multiple pregnancy	
Singleton	5867 (97.2)
Multiple (twin/triplets)	170 (2.8)
Total	6037
Gravidity	
Primigravid	1997 (33.1)
Multigravid	4040 (66.9)
Total	6037
MUAC	
<23 cm	207 (3.4)
≥23 cm	5829 (96.6)
Total	6036
Educational level	
No formal education	308 (5.1)
Some formal education^a^	5729 (94.9)
Total	6037
Nationality	
Ugandan	5954 (98.6)
Other East/Central African	83 (1.40)
Total	6037
HIV status	
HIV seropositive	596 (9.9)
HIV seronegative	5430 (90.1)
Total	6026
Birth outcome	
Stillbirth	60 (1.0)
Live birth	6145 (99.0)
Total	6205
Birthweight	
<2500 g	723 (11.7)
≥2500 g	5477 (88.3)
Total	6200
Gestational age^b^	
Premature (<37 wk)	408 (6.6)
Full term (≥37 wk)	5732 (93.4)
Total	6140

Abbreviations: HIV, human immunodeficiency virus; MUAC, mid-upper arm circumference.

^a^Includes women with primary, secondary, and university-level education.

^b^Based on Ballard score.

### HBsAg Prevalence Among the Study Population

HBsAg results were available for 6012 of the 6037 (99.6%) participants who were tested. Reasons for unavailability of results included participant refusal and insufficient serum for serological testing. The overall proportion of HBsAg positivity was 2.2% (95% CI, 1.8%–2.6%). Only 10 of the 131 (7.6%) study participants who were HBsAg positive were successfully referred to the gastroenterology service for treatment of their infection. Reasons for referral failure included a change or lack of functioning phone contact, refusal to attend for referral, and movement out of the Kampala area. None of the participants had been previously diagnosed with HBV, and none had received prior treatment.

### Factors Associated With HBsAg Positivity

HBsAg-positive and HBsAg-negative women in our study cohort were similar in terms of their age, level of education, gravidity, parity, and nutritional status based on MUAC (Table 2). Of the 591 women who were living with HIV, 20 (3.4%) were also HBsAg positive, greater than the proportion of HIV negative women with that were HBsAg positive (2.1%). This difference was significant on unadjusted analysis (*P* = .04).

**Table 2.** Associations Between the Demographic, Obstetric, and Medical Characteristics of Tested Study Participants and Hepatitis B Surface Antigen Positivity

Characteristic	HBsAg Positive n (row %)	HBsAg Negative n (row %)	Unadjusted RR	(95% CI)	*P* Value
Age					
≤20 y	28 (2.3)	1177 (97.7)	…	…	
>20 y	103 (2.1)	4703 (97.9)	0.9	(.6–1.4)	.7
Gravidity					
Primigravida	43 (2.1)	2007 (97.9)	…	…	
Multigravida	88 (2.2)	3874 (97.8)	1.1	(.7–1.5)	.8
MUAC					
≥23 cm	126 (2.2)	5677 (97.8)	…	…	
<23 cm	5 (2.4)	202 (97.6)	0.9	(.4–2.2)	.8
Educational level					
No formal education	5 (1.6)	302 (98.4)	…	…	
Some formal education	126 (2.2)	5579 (97.8)	1.4	(.6–3.3)	.5
Nationality					
Ugandan	127 (2.1)	5802 (97.9)	…	…	
Other East/Central African	4 (4.8)	79 (95.2)	2.2	(.9–5.9)	.1
HIV status					
Seronegative	111 (2.1)	5299 (98.0)	…	…	
Seropositive	20 (3.4)	571 (96.6)	1.64	(1.0–2.6)	.04^a^
No. of low-birthweight infants delivered					
0	117 (2.2)	5292 (97.8)	…	…	
≥1	14 (2.3)	586 (97.7)	1.2	(.7–2.0)	.5

Data are presented as No. (%) unless otherwise indicated.

Abbreviations: CI, confidence interval; HBsAg, hepatitis B surface antigen; HIV, human immunodeficiency virus; MUAC, mid-upper arm circumference; RR, relative risk.

^a^Indicates statistical significance (*P* < .05).

## DISCUSSION

Our HBsAg prevalence estimate of 2.2% demonstrates intermediate endemicity of HBV infection among our study cohort, defined as an HBV infection prevalence between 2% and 7% [17]. This estimate is lower than Uganda's national HBV infection prevalence estimate of 4.3% [18], but similar to previous estimates among pregnant women within this geographical region. Kayondo et al estimated the prevalence of HBsAg to be 2.9% (95% CI, 1.58%–5.40%) among pregnant women attending another large government-funded hospital in Kampala, Uganda, for antenatal care [19]. An HBsAg prevalence of 2.1% (95% CI, 1.0%–4.1%) was also estimated among pregnant women attending antenatal care in the Lwengo district in southwest Uganda [20]. There is some variation in the reported HBV seroprevalence among pregnant populations across Uganda. A cross-sectional survey conducted in the northern district of Gulu estimated the prevalence of HBsAg among pregnant women at 11.8% [21]. Other studies have shown this region of Uganda to have a high HBV prevalence; a population-based survey conducted in 2013 estimated the prevalence of HBV infection in the Gulu district at 17.6% [22]. Our study estimate is also lower than the pooled prevalence estimate of HBV infection among pregnant woman in Africa of 6.8% (95 CI, 6.1%–7.6%) calculated in a systematic review and meta-analysis by Bigna and colleagues [23].

Of the demographic, obstetric, and medical factors analyzed, only HIV infection was found to be significantly associated with HBsAg positivity within our cohort. The calculated HIV seropositivity prevalence estimate of 15.3% (20/131) among our HBsAg-positive participants was higher than in some of the other estimates from this region. A cross-sectional study conducted in Rwanda by Mutagoma et al estimated the prevalence of HIV infection among HBsAg-positive pregnant women to be 4.1% (95% CI, 2.5%–6.3%) [24]. Another cross-sectional study conducted by Bayo and colleagues in the Gulu district estimated the prevalence of HBV infection among women living with HIV attending antenatal care at 10.8% but found no association between HIV status and HBsAg positivity (10.8% vs 11.9%, *P* = .89) [21], highlighting the perils of relying on this association to identify women who are at greatest risk of HBV infection. Risk-based strategies for HBV testing are generally not recommended, even in low endemicity settings, as there is a risk that a significant proportion of HBV-infected individuals who do not have any identifiable risk factors or do not disclose risk factors due to fear of stigma may go untested [25]. Antenatal screening for HBV should, therefore, be offered to all women attending antenatal care, in line with the WHO's current recommendations for prevention of MTCT of the disease [12].

The low rate of successful onward referral of HBsAg-positive participants to the gastroenterology service at Mulago Hospital, despite pre- and post-test counseling, may be due in part to the stigma that is commonly associated with the diagnosis of HBV infection, particularly in relation to the need for contact tracing and partner testing. It may also reflect a poor understanding of the virus and its associated morbidity among our study cohort. One study examining attitudes to antenatal HBV testing conducted in northern Uganda found that in addition to limited access to HBV testing, other factors associated with poor engagement with antenatal HBV screening services included young maternal age (<25 years), husband's educational level (<7 years of formal education), and low perceived risk of HBV infection [26]. Strategies to improve HBV testing and treatment uptake in this region must, therefore, involve measures to improve health literacy among both men and women [27]. Such measures may include harnessing the influence of civil society organizations within Uganda, such as the National Organisation for People Living with Hepatitis B, to improve knowledge and awareness about the disease within local communities [28]. These organizations and the advocacy they provide play a crucial role in encouraging community members to engage with services, which will be essential in the pursuit of an HBV-free future for Africa [29].

While serological analysis remains the gold standard for the diagnosis of HBV infection, clinical laboratories are highly centralized in Uganda and mainly present in urban areas, with around 96% being privately owned and operated [30]. Antenatal care providers in rural regions may not, therefore, have access to facilities that can perform laboratory-based immunoassays on serum samples collected from pregnant women, nor might they have the equipment available to draw and correctly store these blood samples. Challenges in acquisition of reagents required for laboratory assays, stockouts of supplies, and lack of trained staff are also significant hurdles to ensuring access to HBV screening for all women during pregnancy [31]. Additionally, where women do have access to this level of care, the cost of this test and any subsequent treatment required may be prohibitive for many. The high out-of-pocket cost means many pregnant women are forced to forgo antenatal testing. Some institutions in Uganda offer HBV testing for free [32]; however, this is not commonplace. Point-of-care (POC) testing for HBsAg may provide an inexpensive solution to these challenges. In particular, qualitative POC tests may enable those without laboratory training to interpret test results and initiate a referral for confirmatory testing and management for those with a positive test. A number of HBsAg POC tests have already been validated for use in many parts of Africa and prequalified by the WHO [33], such as the Determine HBsAg POC test (Alere Inc, Waltham, Massachusetts), which has been shown to be acceptable and reliable for the diagnosis of HBV infection among HIV-seronegative women in an antenatal setting in South Africa [34].

Recent work by Nayagam and colleagues showed that antenatal screening for HBsAg and subsequent offer of peripartum antiviral prophylaxis to those who test positive in low- and middle-income countries could be a cost-effective HBV elimination strategy if low-cost diagnostic tests and antiviral medication can be procured [35]. Additionally, the use of multiplex POC tests, which also screen for other blood-borne pathogens such as HIV and syphilis, and the integration of management of these diseases into a single service may prove even more cost-effective in these contexts [36–38]. Innovative financing mechanisms may be needed to eliminate the need for out-of-pocket payments to allow free HBV testing and treatment for all pregnant women [39]. A careful evaluation of diagnostic and treatment patient pathways must accompany this assessment to ensure the sustainability and longevity of antenatal HBV screening programs in this setting—an essential need for elimination of MTCT of this disease.

Our study had some important limitations. First, we did not sample all women attending the labor ward, which may have led to a biased HBsAg prevalence estimate. There may have been factors within the PROGRESS study exclusion criteria that led to the exclusion of women who were at increased risk of HBV infection. For example, participants were excluded from the study if they lived far from the hospital and could not readily return for study follow-up visits, meaning women who lived outside of the city of Kampala were likely excluded. There is ample evidence within the literature that HBV prevalence is typically higher in rural settings compared to urban [40–42]. It is, therefore, possible that the true HBsAg prevalence among the women delivering at this hospital is higher than our estimate and closer to the country-wide prevalence estimate of 4.3%.

Second, we were unable to determine what proportion of infants born to HBsAg-positive women developed HBV infection as we did not test their infants for HBV, so we do not have any specific data about the rate of MTCT within our study cohort. We also have limited data about how many of the infants born to women with HBV infection were vaccinated against HBV. Birth-dose HBV vaccinations are not routinely administered to infants born at Kawempe National Referral Hospital and many of the infants born to women in our cohort did not receive their 6-week vaccinations at the hospital's vaccination clinic. Establishing the current rate of MTCT of HBV in this population would be an important initial step in determining the effectiveness of antenatal HBV screening programs in the prevention of MTCT of HBV in this setting.

Third, the structure and the timings of the PROGRESS study, in which this project was embedded, meant that the HBsAg results could not be returned to the participants prior to the delivery of their infant. Women were recruited while in active labor and blood samples were taken after their infant was born; thus, it was not possible to return their results in time to commence antiviral prophylaxis prior to delivery. Additionally, many of the women who were enrolled into the study did not receive antenatal care at Kawempe National Referral Hospital. These factors were considered during the study design phase and it was decided that the benefits of conducting the HBsAg test outweighed any potential harm caused by the timing of the test. It provided an opportunity for the HBsAg-positive women in our cohort to be made aware of their diagnosis and be referred for specialist care. The study team made every attempt to contact each participant who tested positive for HBsAg to inform them of their result, refer them to the gastroenterology team, and encourage engagement with the Uganda National Extended Programme on Immunization to ensure HBV vaccination for their infant 6 weeks after delivery [43].

## CONCLUSIONS

Our study findings support the urgent need for robust policies that prioritize universal HBV testing among pregnant women and enable those who are eligible to commence antiviral prophylaxis prior to delivery—a strategy that has been found to be cost-effective in similar settings [44]. Antenatal care also provides an opportunity to establish those who are diagnosed with active HBV infection under the care of a specialist for ongoing disease management [11, 27]. Antenatal HBV screening and perinatal antiviral prophylaxis, together with infant immunoprophylaxis, are essential strategies for achieving the goal of elimination of HBV infection by 2030 as outlined in the Sustainable Development Goal 3.3 [12, 45]. Achieving universal antenatal HBV screening will be an important step toward achieving elimination of MTCT of HBV globally [37].

## Notes

***Author contributions.*** K. L. D. and M. E. conceptualized the research. H. G. D., A. A., M. K., V. T., A. N., G. O., G. M., and M. S. were responsible for the conduct of the study and oversight of data collection activities. A. A., J. B., G. O., and M. S. undertook the laboratory analysis. M. E., H. G. D., and K. L. D. analyzed and interpreted the data. M. E., H. G. D., K. L. D., and J. P. drafted the manuscript. All authors contributed to further editing and critical review of the manuscript and approved the final version.

***Acknowledgments.*** We thank the staff at Kawempe National Referral Hospital, as well as all the families and children involved in the study. The complete PROGRESS Study Author Group is comprised of Abdelmajid Djennad, Agnes Nyamaizi, Agnes Ssali, Alexander Amone, Amusa Wamawobe, Annettee Nakimuli, Caitlin Farley, Carol Nanyunja, Christine Najuka, Cleophas Komugisha, Dan R. Shelley, Edward A. R. Portal, Ellie Duckworth, Emilie Karafillakis, Geraldine O’Hara, Godfrey Matovu, Hannah G. Davies, Janet Seeley, Joseph Peacock, Juliet Nsimire Sendagala, Katie Cowie, Kirsty Le Doare, Konstantinos Karampatsas, Lauren Hookham, Madeleine Cochet, Margaret Sewegaba, Mary Kyohere, Maxensia Owor, Melanie Etti, Merryn Voysey, Moses Musooko, Musa Sekikubo, Owen B. Spiller, Patience Atuhaire, Paul T. Heath, Philippa Musoke, Phiona Nalubega, Pooja Ravji, Richard Katungye, Ritah Namugumya, Rosalin Parks, Rose Azuba, Sam Kipyeko, Simon Beach, Stephen Bentley, Tim Old, Tobius Mutabazi, Valerie Tusubira, and Vicki Chalker.

***Patient consent.*** Written informed consent was obtained from all patients involved in the study.

***Ethical approval.*** The study has been approved by the Ugandan National Council for Science and Technology (reference number HS 2496); the Makerere University School of Medicine Research and Ethics Committee (SOMREC; 2018–130); and St George's, University of London (SGUL REC reference nynber 2020.0024), who sponsored the study. The protocol was published in Gates Open Research in June 2020 [14].

***Data availability.*** The full dataset for the PROGRESS study is available at doi:10.24376/rd.sgul.24101115.v1 [46].

***Disclaimer.*** The content of the manuscript is solely the responsibility of the authors. The funders had no role in study design, data collection and analysis, decision to publish, or preparation of the manuscript.

***Financial support*.** This study was funded by the Bill & Melinda Gates Foundation (grant number OPP1189053). This work was also supported by the National Institute for Healthcare Research for the qualitative research (16/136/46) and sponsored by St George's, University of London (R&D protocol code number 17.0018).

***Supplement sponsorship.*** This article appears as part of the supplement “Neonatal and Young Infant Infection in Uganda: the PROGRESS Study,” sponsored by St George's, University of London.
